# Sotrovimab versus usual care in patients admitted to hospital with COVID-19 (RECOVERY): a randomised, controlled, open-label, platform trial

**DOI:** 10.1016/S1473-3099(25)00361-5

**Published:** 2025-08-28

**Authors:** RECOVERY Collaborative Group, Peter W Horby, Jonathan R Emberson, Leon Peto, Natalie Staplin, Mark Campbell, Guilherme Pessoa-Amorim, Richard Stewart, Dipansu Ghosh, Graham Cooke, Natalie Blencowe, Jeronimo Moreno-Cuesta, Purav Desai, Paul Hine, Jonathan Underwood, Nicholas Easom, Jaydip Majumdar, Sanjay Bhagani, J Kenneth Baillie, Maya H Buch, Saul N Faust, Thomas Jaki, Katie Jeffery, Edmund Juszczak, Marian Knight, Wei Shen Lim, Alan Montgomery, Aparna Mukherjee, Andrew Mumford, Kathryn Rowan, Guy Thwaites, Marion Mafham, Richard Haynes, Martin J Landray

## Abstract

**Background:**

Sotrovimab is a neutralising monoclonal antibody targeting the SARS-CoV-2 spike protein. We aimed to evaluate the efficacy and safety of sotrovimab in the RECOVERY trial, an investigator-initiated, individually randomised, controlled, open-label, adaptive platform trial testing treatments for patients admitted to hospital with COVID-19.

**Methods:**

Patients admitted with COVID-19 pneumonia to 107 UK hospitals were randomly assigned (1:1) to either usual care alone or usual care plus a single 1 g infusion of sotrovimab, using web-based unstratified randomisation. Participants were eligible if they were aged at least 18 years, or aged 12–17 years if weighing at least 40kg, and had confirmed COVID-19 pneumonia with no medical history that would put them at significant risk if they participated in the trial. Participants were retrospectively categorised as having a high antigen level if baseline serum SARS-CoV-2 nucleocapsid antigen was above the median concentration (the prespecified primary efficacy population), otherwise they were categorised as having a low antigen level. The primary outcome was 28-day mortality assessed by intention to treat. Safety outcomes were assessed among all participants, regardless of antigen level. Recruitment closed on March 31, 2024, when funding ended. The trial is registered with ISRCTN (50189673) and ClinicalTrials.gov (NCT04381936).

**Findings:**

From Jan 4, 2022, to March 19, 2024, 1723 patients were enrolled in the RECOVERY sotrovimab comparison. Of these, 828 (48%) were assigned to usual care plus sotrovimab and 895 (52%) were assigned to usual care only. Mean patient age was 70·7 years (SD 14·8) and 1033 (60%) were male. 720 (42%) patients were classified as having a high antigen level, 717 (42%) as having a low antigen level, and 286 (17%) had unknown antigen status. 1389 (81%) patients were vaccinated, 1179 (82%) of 1438 patients with known serostatus had anti-spike antibodies at randomisation, and 1021 (>99%) of 1026 patients with sequenced samples were infected with omicron variants. Among patients with a high antigen level, 82 (23%) of 355 assigned to sotrovimab versus 106 (29%) of 365 assigned usual care died within 28 days (rate ratio 0·75, 95% CI 0·56–0·99; p=0·046). In an analysis of all randomly assigned patients (regardless of antigen status), 177 (21%) of 828 patients assigned to sotrovimab versus 201 (22%) of 895 assigned to usual care died within 28 days (0·95, 0·77–1·16; p=0·60). Infusion reactions were recorded in 12 (2%) of 781 patients receiving sotrovimab. We found no difference between groups in any other safety outcome.

**Interpretation:**

In patients admitted to hospital with COVID-19 pneumonia, sotrovimab was associated with reduced mortality in the primary analysis population who had a high serum SARS-CoV-2 antigen concentration at baseline, but not in the overall population. Treatment options for patients admitted to hospital are limited, and mortality in those receiving current standard of care was high. The emergence of high-level resistance to sotrovimab among subsequent SARS-CoV-2 variants restricts its current usefulness, but these results indicate that targeted neutralising antibody therapy could potentially still benefit some patients admitted to hospital who are at high risk of death in an era of widespread vaccination and omicron infection.

**Funding:**

UK Research and Innovation (Medical Research Council) and National Institute for Health and Care Research.

## Introduction

Treatment with neutralising monoclonal antibodies targeting the SARS-CoV-2 spike protein has been found to substantially reduce the risk of admission to hospital or death in patients with early COVID-19 who are at high risk of complications.^1–3^ Neutralising monoclonal antibodies were also found to reduce the risk of death among patients admitted to hospital, but this benefit was restricted to those who had not yet developed their own anti-SARS-CoV-2 antibody response (ie, those who are seronegative).^4–6^ The RECOVERY casirivimab–imdevimab comparison, which recruited patients from Sept 18, 2020, to May 22, 2021, is the largest randomised evaluation of neutralising monoclonal antibody therapy in patients admitted to hospital with COVID-19. In this comparison, 28-day mortality in patients who were seronegative at baseline was double that of patients who were seropositive (30% *vs* 15%), and combination therapy with casirivimab and imdevimab reduced 28-day mortality in patients who were seronegative at baseline to 24% compared with usual care without neutralising monoclonal antibody therapy (rate ratio 0·79, 95% CI 0·69–0·91; p=0·0009).^4^ Following the publication of this study, targeted monoclonal antibody therapy for seronegative patients admitted to hospital with COVID-19 was adopted into routine practice in the UK and elsewhere.

A major limitation of monoclonal antibody therapy has been the frequent emergence of new SARS-CoV-2 variants that are not effectively neutralised by existing antibodies.^7,8^ When the first omicron variant, BA.1, became globally dominant in December, 2021, it contained spike mutations conferring high-level resistance to most monoclonal antibodies in clinical use, including the casirivimab–imdevimab combination, leading to its withdrawal from guidelines.^8^ Sotrovimab, a neutralising monoclonal antibody originally derived from a patient who had recovered from SARS-CoV-1, targets a relatively conserved spike protein epitope. In the COMET-ICE trial of patients with early infection conducted in 2020–21, sotrovimab reduced the risk of hospital admission or death by 79% (adjusted relative risk 0·21, 95% CI 0·09–0·50).^1^ The neutralisation potency of sotrovimab was modestly reduced against BA.1 compared with wild-type virus (approximately 3–5 fold in most studies), but it retained more activity than many other neutralising monoclonal antibodies, which made it a promising candidate for continued use in patients admitted to hospital and prompted its evaluation in RECOVERY.^9,10^ A further reduction in activity against omicron subvariant BA.2 led to the withdrawal of sotrovimab by the US Food and Drug Administration Emergency Use Authorization in April, 2022. However, sotrovimab retained enough in vitro activity against viral variants prevalent in 2022–23 to suggest it could retain clinical benefit via direct neutralisation (because serum sotrovimab concentrations remained around 100 times the 50% effective concentration for BA.2), or via Fc-dependent effector mechanisms.^11,12^ During November, 2023, omicron subvariants BA.2.86 and JN.1 became dominant in the UK and elsewhere, which have an additional spike gene mutation that confers high-level resistance to sotrovimab.^13^

The current role of therapeutic neutralising mono-clonal antibodies in patients admitted to hospital with COVID-19 is also complicated by increasing population immunity to SARS-CoV-2, because the previous trials that identified a benefit in patients who were seronegative were done before widespread vaccination and natural immunity. By the time BA.1 emerged, most people admitted to hospital in the UK with COVID-19 had been vaccinated and many had been previously infected. In this setting, patients would be expected to have detectable anti-SARS-CoV-2 antibodies at admission, but this could reflect immune responses to previous vaccination or infection that were insufficient to prevent the current illness, rather than adaptive immunity to the current infection. This ambiguity regarding the source of antibodies suggests that biomarkers of infection other than serostatus might now be required to identify which, if any, patients admitted to hospital could benefit from neutralising monoclonal antibody treatment.

One possible biomarker is SARS-CoV-2 antigenaemia. Viral nucleocapsid antigen is detectable in the blood of most patients admitted to hospital with COVID-19, and high concentrations are strongly correlated with more severe disease and worse prognosis.^14–16^ In most patients admitted to hospital with COVID-19, antigen concentrations fall rapidly in the first few days of admission as they clear the infection.^17^ The degree of antigenaemia is inversely correlated with specific antibody responses, but, unlike antibodies, detection of viral antigen is highly likely to be related to the current infection.

Here, we report the results of the sotrovimab comparison in RECOVERY, a randomised, controlled, open-label platform trial evaluating treatments for patients admitted to hospital with COVID-19 pneumonia. Recruitment occurred in the UK during a period in which omicron variants were dominant and most people were vaccinated against SARS-CoV-2.

## Methods

### Study design and participants

The Randomised Evaluation of COVID-19 Therapy (RECOVERY) trial is an investigator-initiated, individually randomised, controlled, open-label, adaptive platform trial to evaluate the effects of potential treatments in patients admitted to hospital with COVID-19. Details of the trial design and results for other treatments have been published previously and are available on the study website.^18^ The trial was conducted at hospital organisations in the UK and is supported by the National Institute for Health and Care Research (NIHR) Clinical Research Network. 107 hospitals in the UK enrolled participants in the sotrovimab comparison (appendix pp 5–32). The trial is coordinated by the Nuffield Department of Population Health at the University of Oxford (Oxford, UK), the trial sponsor. It is conducted in accordance with the principles of the International Conference on Harmonisation–Good Clinical Practice guidelines and is approved by the UK Medicines and Healthcare products Regulatory Agency and the Cambridge East Research Ethics Committee (ref: 20/EE/0101). The protocol, statistical analysis plan, and additional information are available in the appendix (pp 71–200) and on the study website.

Patients admitted to hospital were eligible for the study if they had confirmed SARS-CoV-2 infection (by PCR or lateral flow test) with a pneumonia syndrome thought to be related to COVID-19, and no medical history that might, in the opinion of the managing physician, put the patient at significant risk if they were to participate in the trial. Patients were excluded from the sotrovimab comparison if they were younger than 12 years or were younger than 18 years and weighed less than 40 kg. Women who were pregnant or breastfeeding were eligible. Written informed consent was obtained from all patients, or a legal representative if patients were too unwell or otherwise unable to provide informed consent.

### Randomisation and masking

Eligible and consenting patients were randomly assigned in a 1:1 ratio to either usual standard of care plus sotrovimab or usual standard of care alone, using web-based simple (unstratified) randomisation with allocation concealed until after randomisation (appendix pp 42–44).

As a platform trial, and in a factorial design, patients could be simultaneously included in other concurrently evaluated treatment comparisons, each having its allocation established by an independent 1:1 randomisation for: (1) empagliflozin versus usual care, (2) higher-dose corticosteroids versus usual care, (3) molnupiravir versus usual care, and (4) nirmatrelvir–ritonavir versus usual care (appendix pp 42–43). Participants and local study staff were not masked to the allocated treatment. Other than members of the RECOVERY data monitoring committee, all individuals involved in the trial were masked to aggregated outcome data while recruitment and 28-day follow-up were ongoing.

### Procedures

Baseline data were collected using a web-based case report form that included demographics, level of respiratory support, major comorbidities, suitability of the study treatment for a particular patient, SARS-CoV-2 vaccination status, and study treatment availability at the study site. Sex was recorded as male, female, or unknown, based on hospital records (appendix p 47). A serum sample and nose swab were collected at randomisation and sent to central laboratories for testing. Serum was tested for SARS-CoV-2 nucleocapsid antigen, anti-SARS-CoV-2 spike antibodies, and anti-SARS-CoV-2 nucleocapsid antibodies using Roche Elecsys assays (Roche Diagnostics, Basel, Switzerland). Patients were classified as having a high or low serum nucleocapsid antigen level using the trial population median value (cutoff index 0·626, corresponding to a nucleocapsid protein concentration of 12·5 pg/mL; appendix p 191), and as positive or negative for anti-spike and anti-nucleocapsid antibodies using manufacturer defined thresholds (testing was retrospective, so results were not available to the patient’s medical team). Nose swabs were tested for SARS-CoV-2 RNA using TaqPath COVID-19 RT-PCR (Thermo Fisher Scientific, MA, USA). Samples with sufficient viral RNA were sequenced using the SARS-CoV-2: Midnight Protocol (Oxford Nanopore Technologies, Oxford, UK).^19^ Sequence data were used to detect spike protein mutations associated with a more than five-fold reduction in sotrovimab neutralisation, which were identified from the sotrovimab summary of product characteristics and the Stanford University Coronavirus Antiviral & Resistance Database.^20^ Further details of laboratory analyses and the resistance mutations included are in the appendix (pp 33–34, 184–201).

Follow-up nose swabs were collected on day 3 and day 5 (counting the day of randomisation as day 1). These swabs were analysed in the same manner as the baseline swab described.

Patients allocated to sotrovimab were to receive 1 g in 100 mL 0·9% saline or 5·0% glucose intravenously over 60 min as soon as possible after randomisation. This dose of sotrovimab is double the licensed dose for early infection and was selected because of reduced neutralisation activity against BA.1 compared with wild-type virus. All other aspects of patient care were to be decided by the managing clinician in line with their usual practice.

An online follow-up form was completed when participants were discharged, had died, or 28 days after randomisation, whichever occurred earliest (appendix pp 48–56). Information was recorded on adherence to allocated study treatment, receipt of other COVID-19 treatments, duration of admission, receipt of respiratory or renal support, major safety outcomes, and vital status (including cause of death). In addition, routine health-care and registry data were obtained, including information on vital status (with date and cause of death), discharge from hospital, receipt of respiratory support, or renal replacement therapy.

### Outcomes

Outcomes were assessed at 28 days after randomisation, with further analyses specified at 6 months after randomisation (not reported here). The primary outcome was all-cause mortality at 28 days. Secondary outcomes were time to discharge from hospital, and, among patients who were not on invasive mechanical ventilation at randomisation, the composite outcome of invasive mechanical ventilation (including extracorporal membrane oxygenation) or death. Prespecified subsidiary clinical outcomes were use of invasive or non-invasive ventilation (including high-flow nasal oxygen) among patients who were not on any ventilation at randomisation, and use of renal dialysis or haemofiltration. Prespecified safety outcomes were cause-specific mortality, major cardiac arrhythmia, thrombotic and major bleeding events, non-SARS-CoV-2 infections, hyperglycaemia or hypoglycaemia, seizures, acute liver or kidney injury, and infusion reactions to sotrovimab. Virological outcomes were viral RNA copy number in nose swabs taken at day 3 and day 5, and the frequency of detection of resistance mutations. Information on suspected serious adverse reactions was collected in an expedited fashion to comply with regulatory requirements. Details of the methods used to ascertain and derive outcomes are provided in the appendix (pp 160–83).

### Statistical analysis

Because trial recruitment and event rates during the COVID-19 pandemic were unpredictable, RECOVERY treatment comparisons did not have a predetermined sample size. With high levels of recruitment, the intention would have been to continue until enough primary outcomes had accrued for a 90% power to detect a proportional risk reduction of 20% at a two-sided p value of 0·01 (approximately 5500 participants if mortality were 20% without treatment). Following the initial wave of omicron infection in the UK in the first half of 2022, the number of patients admitted to hospital with COVID-19 pneumonia reduced substantially in the UK, as did trial recruitment. The trial comparison closed on March 31, 2024, when funding for the trial ended.

For all outcomes, we did intention-to-treat analyses comparing patients randomly assigned to sotrovimab with patients who were randomly assigned to usual care. For the primary outcome of 28-day mortality, the hazard ratio from a Cox model with adjustment for age in three categories (<70 years, 70–79 years, and ≥80 years) and ventilation status at randomisation in four categories (no oxygen, simple oxygen only, non-invasive ventilation, and invasive mechanical ventilation) was used to estimate the mortality rate ratio. We constructed Kaplan–Meier survival curves to display cumulative mortality over the 28-day period (starting on the day of randomisation and ending 28 days later). We used the same Cox regression method to analyse time to hospital discharge and successful cessation of invasive mechanical ventilation, with patients who died in hospital right-censored on day 29. There was no evidence against the proportionality assumption for the primary outcome of 28-day mortality. Safety outcomes were assessed among all participants, regardless of antigen level

Median time to discharge was derived from Kaplan–Meier estimates. For the composite secondary outcome of progression to invasive mechanical ventilation or death within 28 days, and the subsidiary clinical outcomes of receipt of ventilation and use of haemodialysis or haemofiltration, the precise dates were not available, and a log-binomial regression model was used to estimate the risk ratio (RR) adjusted for age and ventilation status (in the same categories as previously listed). Estimates of rate and RRs are shown with 95% CIs. SARS-CoV-2 viral RNA levels in nose swabs were estimated with analysis of covariance (ANCOVA) using the log-transformed values after adjustment for each participant’s baseline value of SARS-CoV-2 viral RNA level, age, and level of respiratory support at randomisation. Missing baseline and follow-up values of SARS-CoV-2 viral RNA levels were estimated using multiple imputation, with 20 replicate sets and combination of results across sets using the methods of Rubin.^21^ For the resistance mutation frequency outcome, we report absolute numbers.

When the sotrovimab comparison was added to the protocol in December, 2021, there was insufficient information to decide whether anti-spike or anti-nucleocapsid antibody status should define the primary analysis population, or if serum antigen status would be preferable. The statistical analysis plan stated that this would be determined at a future date (but before the investigator team was unmasked to treatment allocation). Shortly after recruitment closed, but before the investigators were unmasked, patients with a high antigen level were selected as the primary analysis population because of low numbers of seronegative patients in the trial population and because antigen positivity best predicted mortality (described in the updated statistical analysis plan; appendix pp 151–153). It was hypothesised that any beneficial effect of sotrovimab would be larger among patients with a high antigen level and might be negligible in patients with a low antigen level. Formal hypothesis-testing of the effect of allocation to sotrovimab on 28-day mortality was to be done first in participants with a high antigen level (the primary analysis population), and was to be done among all randomised participants only if a reduction in mortality in patients with a high antigen level was seen at a two-sided p value of less than 0·05. Formal testing of secondary outcomes was only to be done if a mortality reduction among all participants was seen at a two-sided p value of less than 0·05. A prespecified comparison of the effects of allocation to sotrovimab on 28-day mortality in patients with a high antigen level versus those with a low antigen level was done by performing a test for heterogeneity. Tests for heterogeneity according to other baseline characteristics were also prespecified (age, sex, ethnicity, level of respiratory support, days since symptom onset, use of corticosteroids, anti-SARS-CoV-2 antibody status, and immunodeficiency), and a post-hoc analysis of heterogeneity according to use of remdesivir at baseline was performed (appendix p 68).

The full database is held by the study team, which collected the data from study sites and performed the analyses at the Nuffield Department of Population Health at the University of Oxford. Analyses were performed using SAS version 9.4 and R version 4.0.3. The trial is registered with ISRCTN (50189673) and ClinicalTrials. gov (NCT04381936).

### Role of the funding source

Neither the study funders, nor the manufacturers of sotrovimab, had any role in study design, data collection, data analysis, or writing of the report. GSK and Vir Biotechnology supported the study through supply of sotrovimab and reviewed the draft publication for scientific consistency and completeness.

## Results

**Figure 1:**
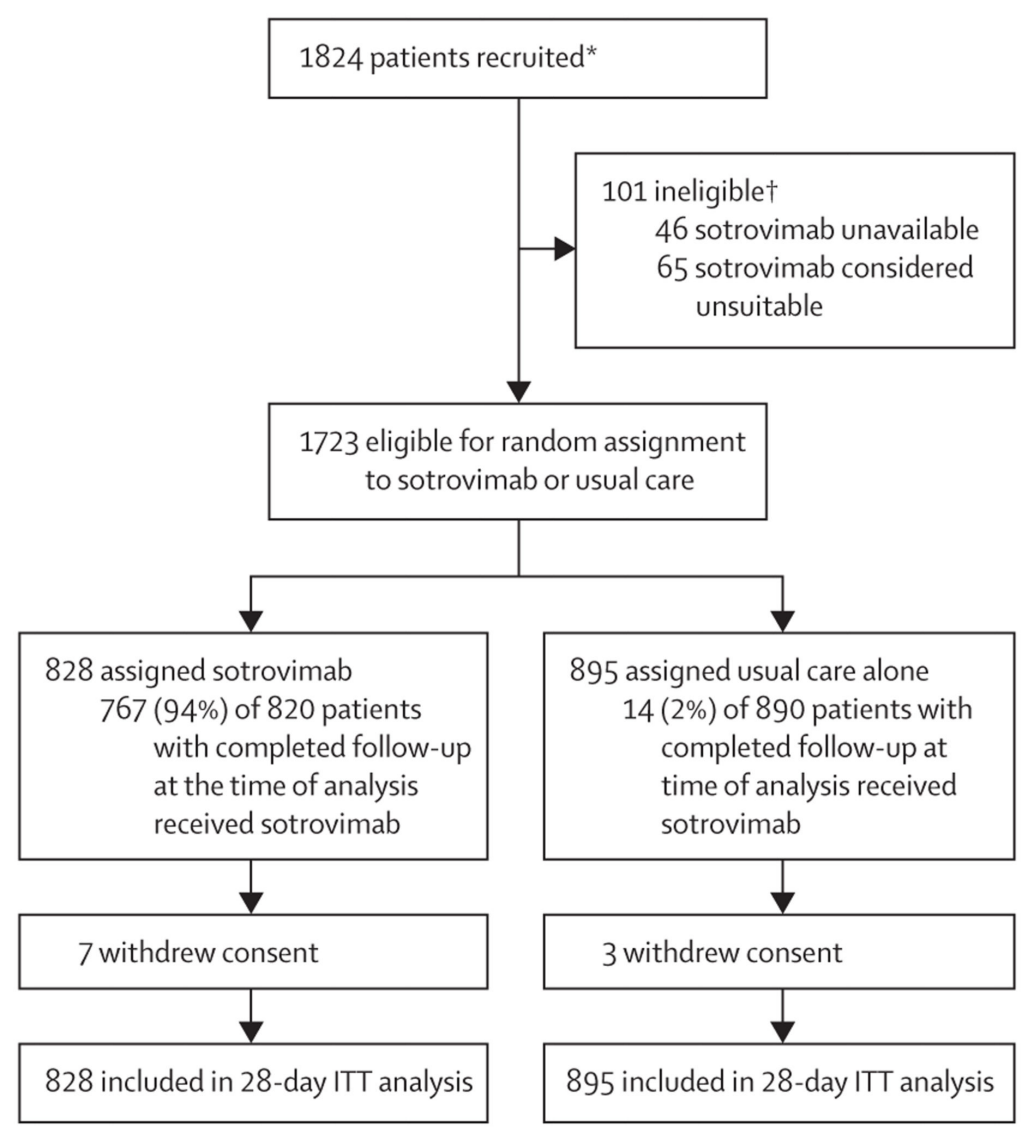
Trial profile ITT=intention to treat. *Number recruited to any RECOVERY comparison at sites participating in the sotrovimab comparison, during the period in which it was open. †Drug unavailability and unsuitability are not mutually exclusive.

Between Jan 4, 2022, and March 19, 2024, 1723 (94%) of 1824 patients enrolled into the RECOVERY trial at sites participating in the sotrovimab comparison were eligible and agreed to participate in the sotrovimab comparison. Of these 1723 eligible participants, 828 (48%) were allocated to sotrovimab and 895 (52%) were allocated to usual care without sotrovimab (figure 1). The mean age of study participants in this comparison was 70·7 years (SD 14·8), 1033 (60%) participants were male, and 690 (40%) participants were female. 1389 (81%) participants had received a COVID-19 vaccine and 414 (24%) were severely immunocompromised in the opinion of the managing clinician (table 1; appendix pp 59–60). At randomisation, the median time since symptom onset was 6 days (IQR 3–11), 1467 (85%) participants were receiving oxygen or ventilatory support, and 628 (36%) were receiving remdesivir. Serological results were available for 1439 (84%) patients, among whom 720 (50%) had a serum nucleocapsid antigen concentration above the median (high antigen level), 1179 (82%) were anti-SARS-CoV-2 spike antibody positive, and 454 (32%) were anti-SARS-CoV-2 nucleocapsid antibody positive (table 1). Baseline serum nucleocapsid antigen level was only moderately correlated with anti-spike and anti-nucleocapsid antibody level (Pearson correlation coefficients –0·37 and –0·22, respectively, using log transformed values).

The follow-up form was completed for 1710 (99%) patients, and among them 767 (94%) of 820 patients allocated to sotrovimab received the treatment, compared with 14 (2%) of 890 allocated to usual care (figure 1). Use of other treatments for COVID-19 was similar among patients allocated sotrovimab and those allocated usual care (appendix p 61).

**Figure 2:**
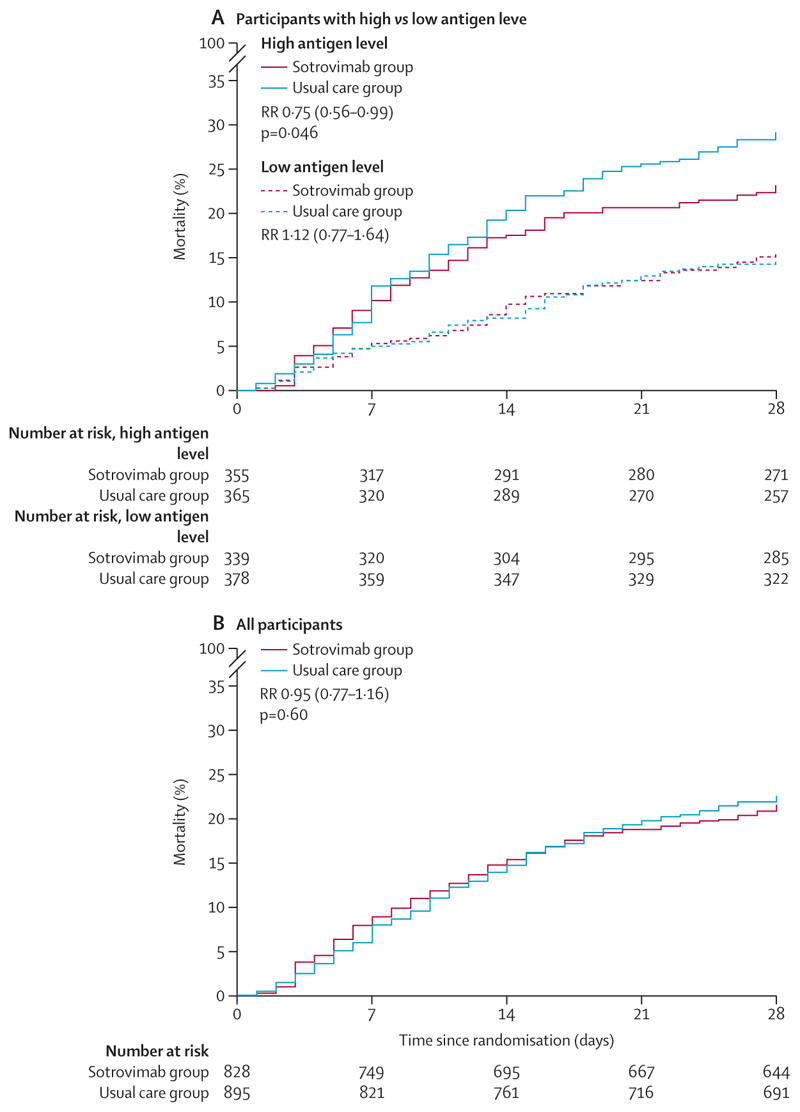
Effect of allocation to sotrovimab on 28-day mortality (A) Patients with a high versus low antigen level. (B) All randomly assigned patients. RR=risk ratio.

**Figure 3:**
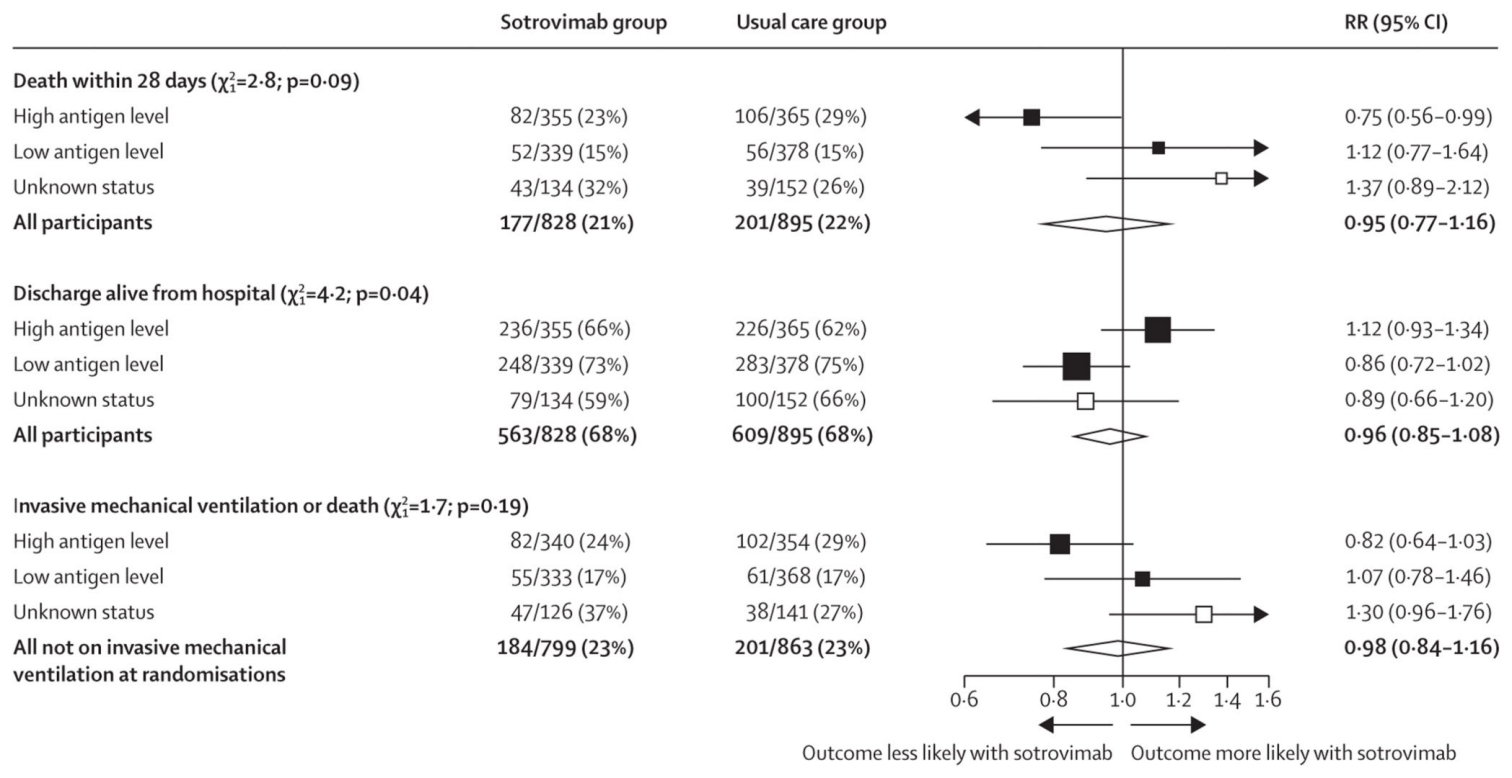
Primary and secondary outcomes, overall and by baseline antigen status Subgroup-specific RR estimates are represented by squares (with areas of the squares proportional to the amount of statistical information) and the lines through them correspond to the 95% CIs. Open squares represent participants with unknown status and solid squares represent participants with known status. The $\chi_{1}^{2}$ tests for heterogeneity compare the log RRs in patients with a high antigen level versus those with a low antigen level (ie, excluding those with unknown antigen status). All participants are included in the overall summary diamonds. RR=risk ratio for the composite outcome of invasive mechanical ventilation or death, and rate ratio for the other outcomes.

**Figure 4:**
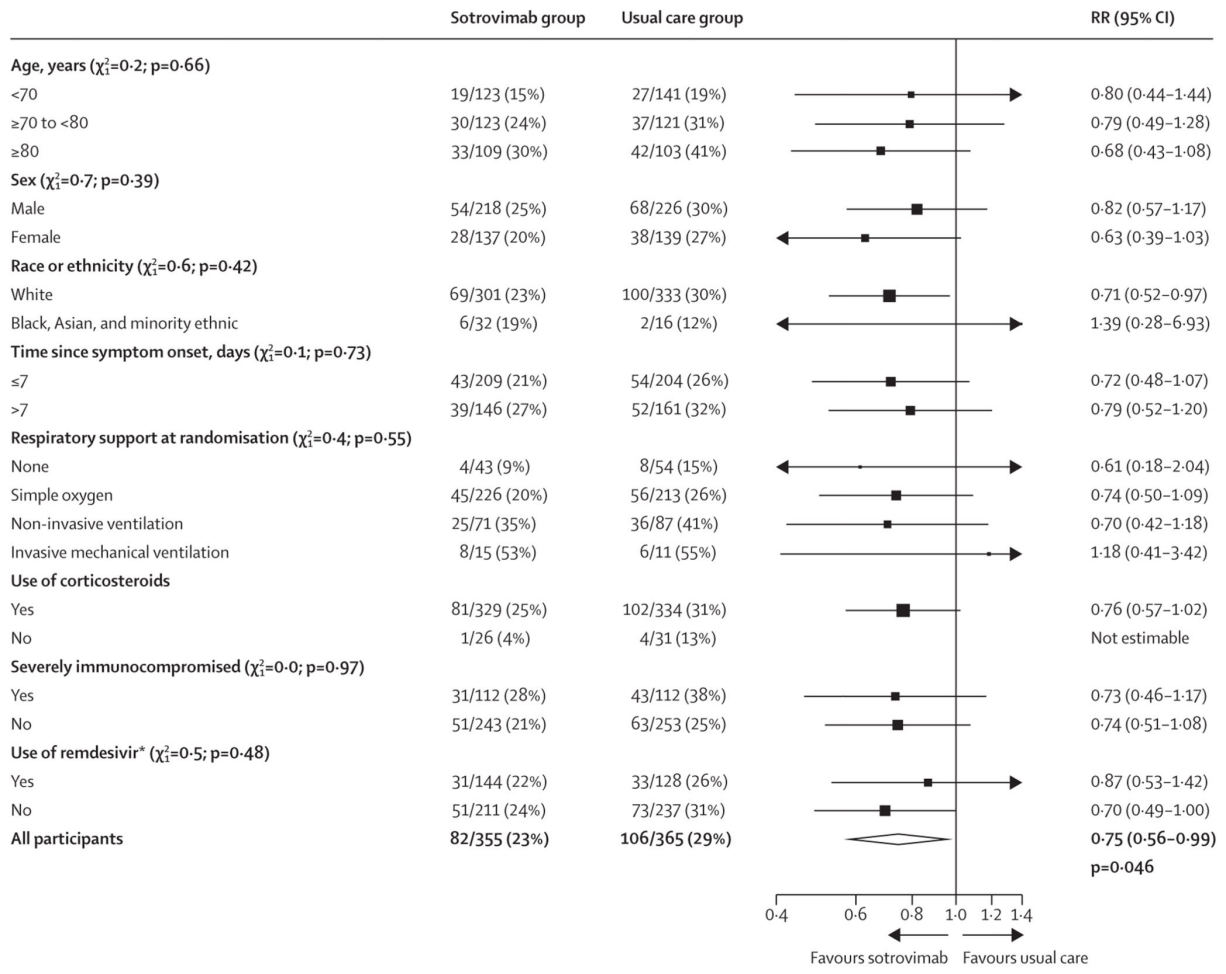
Effect of allocation to sotrovimab on 28-day mortality by baseline characteristics in participants with a high antigen level Subgroup-specific RR estimates are represented by squares (with areas of the squares proportional to the amount of statistical information) and the lines through them correspond to the 95% CIs. The ethnicity subgroup excluded those with missing data, but these patients are included in the overall summary diamond. RR=rate ratio. *Post-hoc subgroup analysis requested during peer review. $\chi_{1}^{2}$ test for heterogeneity or trend.

In patients who had a high antigen level at baseline, allocation to sotrovimab was associated with a reduction in the primary outcome of 28-day mortality compared with usual care alone: 82 (23%) of 355 patients in the sotrovimab group died compared with 106 (29%) of 365 patients in the usual care group (RR 0·75, 95% CI 0·56–0·99; p=0·046; table 2, figures 2A, 3). Among all patients who were randomly assigned (including those with high, low, or unknown baseline antigen level), there was no significant difference in the primary outcome of 28-day mortality between the two treatment groups: 177 (21%) of 828 patients in the sotrovimab group died compared with 201 (22%) of 895 patients in the usual care group (RR 0·95, 0·77–1·16; p=0·60; figures 2B, 3, appendix p 62). There was no evidence that the proportional effects on mortality differed in any pre-specified subgroups or in the post-hoc subgroup analysis of patients receiving remdesivir at baseline, either among patients with a high antigen level or among all patients (figure 4; appendix pp 68–70).

Among patients with a high antigen level, time to discharge from hospital within 28 days did not differ significantly between those allocated to sotrovimab compared with those allocated to usual care (236 [66%] *vs* 226 [62%]; RR 1·12, 95% CI 0·93–1·34; median time to being discharged alive 13 days [IQR 7 to >28] *vs* 16 days [7 to >28]; table 2, figure 3). There was also no difference in this outcome among the overall study population (563 [68%] *vs* 609 [68%]; 0·96, 0·85–1·08; 11 days [IQR 6 to >28] *vs* 11 days [6 to >28]; figure 3; appendix p 62).

Among patients with a high antigen level who were not on invasive ventilation at baseline, allocation to sotrovimab was not associated with a significantly lower risk of progressing to the composite secondary outcome of invasive ventilation or death (82 [24%] of 340 patients *vs* 102 [29%] of 354 patients, RR 0·82, 95% CI 0·64–1·03; table 2, figure 3). There was also no difference in this outcome among the overall study population (184 [23%] of 799 patients *vs* 201 [23%] of 863 patients, 0·98, 0·84–1·16; figure 3; appendix p 62).

We found no evidence of any difference between groups in the prespecified subsidiary outcomes among patients with a high antigen level, or among all patients, including in use of ventilation in those not on ventilation at baseline, successful cessation of ventilation, or use of renal replacement therapy (table 2; appendix p 62).

1479 (86%) of 1723 patients had at least one nose swab available for analysis. Allocation to sotrovimab was not associated with a lower baseline-adjusted viral RNA copy number in nose swabs taken on day 3 or day 5 (table 2). 1119 (65%) of 1723 patients had at least one successfully sequenced sample (≥50% genome coverage), of whom 1114 (>99%) were infected with omicron variants (primarily BA.1, BA.2, BA.5, and XBB). 1655 (96%) of 1723 patients were recruited before Nov 1, 2023, and among these patients, 14 (1%) of 1084 with a sequenced sample had a sotrovimab resistance mutation detected at baseline, and three (<1%) of 692 patients with sequenced baseline and follow-up samples had a new sotrovimab resistance mutation arising after trial entry, two of whom had received sotrovimab (details of these three patients are in the appendix p 65). 68 (4%) of 1723 patients were recruited after Nov 1, 2023, and among these patients, 14 (40%) of 35 with a sequenced sample were infected with BA.2.86 variants, which contain the lineage-defining K356T spike mutation associated with high-level sotrovimab resistance.

12 (2%) of 781 patients who received sotrovimab had an infusion reaction. Of these 12 patients, nine did not require any intervention, two required antihistamines or steroids only, and one required adrenaline. Two of these infusion reactions were reported as serious adverse reactions, both of which resolved, including one episode of anaphylaxis. No other serious adverse reactions to sotrovimab were reported. We found no difference between groups in other safety outcomes, including cause-specific mortality, new cardiac arrhythmia, thrombosis, bleeding, non-coronavirus infections, hypoglycaemia or hyperglycaemia, seizures, acute kidney injury, or liver injury (appendix pp 63–64).

## Discussion

In this randomised trial including 1723 patients with COVID-19 pneumonia, sotrovimab was associated with a reduction in 28-day mortality in those with a high serum nucleocapsid antigen level when compared with usual care, although there was substantial uncertainty about the size of this apparent benefit (RR 0·75, 95% CI 0·56–0·99; p=0·046). An analysis of all patients, regardless of antigen concentration, did not show evidence of any significant benefit of treatment with sotrovimab on 28-day mortality. In contrast with our previous study of neutralising monoclonal antibody treatment in patients admitted to hospital, the current study was performed during a period of omicron variant infection and widespread vaccination and natural immunity, making it more relevant to the treatment of current and future patients admitted to hospital with COVID-19.^4^

The number of patients admitted to hospital with COVID-19 pneumonia fell dramatically after vaccination was introduced and omicron became dominant, so this comparison could not provide results that are as definitive as those of the earlier RECOVERY casirivimab–imdevimab comparison, which recruited nearly 10 000 patients. However, the pattern of results from the two RECOVERY monoclonal antibody comparisons are similar, despite using different markers of infection status to categorise patients. In both comparisons, a subset of patients with immune responses that were not yet adequate to clear infection were at higher risk of death than patients with more robust immune responses. Furthermore, in both subsets at higher risk of death, monoclonal antibody therapy reduced the risk of death.

During the period that the sotrovimab comparison was recruiting, SARS-CoV-2 infection was often an incidental finding in patients admitted to hospital, or was associated with non-respiratory illness, and it is possible that these patients would derive less benefit from antiviral treatment that the patients included in RECOVERY, who required COVID-19 pneumonia for trial entry. In 1389 (81%) of participants, COVID-19 pneumonia had developed despite previous COVID-19 vaccination. In keeping with this, 1179 (82%) of 1438 of those with known serostatus had anti-spike antibodies, although 985 (68%) of 1439 were anti-nucleocapsid antibody negative, indicating that this was probably their first SARS-CoV-2 infection.^22^ The risk of death from COVID-19 was high, despite standard supportive care and the availability of immunomodulation and antiviral treatment with remdesivir. 28-day mortality was 22% among all patients allocated to usual care, which was similar to the risk among RECOVERY patients recruited in the pre-omicron era.^4^ Since the emergence of omicron, patients who are immunocompromised have made up a higher proportion of those admitted to hospital for, and dying from, COVID-19 pneumonia; indeed, 414 (24%) of 1723 patients in this RECOVERY comparison were considered severely immunocompromised.^23^ Current treatment options for patients admitted to hospital with COVID-19 are insufficient, particularly for patients who are immuno-compromised, in whom immunomodulatory therapies should be used with caution.^24^ Our results indicate that targeted neutralising antibody therapy could potentially still provide benefit for certain patients at high risk of death, even when administered more than a week after symptom onset.

The benefit of monoclonal antibody therapy in patients admitted to hospital who are negative for SARS-CoV-2 antibodies was established in previous trials, but this approach to targeting therapy was necessarily short-lived in the context of increasing population immunity.^4–6^ By contrast, targeting therapy on the basis of antigenaemia remains possible for future patients who are admitted to hospital, and is practical using existing commercial assays; the assay used in RECOVERY takes 20 min on a widely available automated clinical laboratory platform. To our knowledge, the ACTIV-3/TICO platform trial is the only previous trial of monoclonal antibody therapy reporting outcomes by baseline blood antigen status, and this trial evaluated four monoclonal antibody therapies, although three of these were stopped early for futility.^6,14,25^ In the only comparison that was not stopped early, 1417 patients admitted to hospital were randomly assigned to receive combined tixagevimab and cilgavimab or placebo. Among patients with blood antigen concentrations above the median value, 90-day mortality was 13% (43 of 340) in those assigned to monoclonal antibody treatment versus 15% (51 of 342) in those assigned to placebo (hazard ratio 0·84, 95% CI 0·56–1·26; p=0·39). Although inconclusive, the point estimate from the ACTIV-3/TICO trial is consistent with this RECOVERY result that is based on twice as many events. Compared with blood antigen and antibody concentrations, the quantity of viral RNA collected when sampling the upper respiratory tract is highly variable, even in simultaneously collected swabs.^26^ This variability restricts its usefulness as a marker to predict an individual’s treatment response, so subgroup analyses by nasal RNA viral copy number were not performed in this sotrovimab comparison.

Neutralising monoclonal antibodies emerged as powerful therapeutic tools during the pandemic, which has highlighted their potential uses but also their limitations, particularly the loss of activity against emergent viral variants. Despite retaining potentially valuable neutralising activity against omicron variants prevalent in 2022–23, high-level sotrovimab resistance was identified in omicron lineages that became globally dominant in early 2024, including BA.2.86 and JN.1, and it is no longer likely to have useful activity against currently circulating variants that have retained sotrovimab resistance mutations.^27^ The loss of all anti-SARS-CoV-2 monoclonal antibodies that were in clinical use has led to new approaches to monoclonal antibody therapy, including attempts to target more highly conserved viral epitopes, new antibody fragments or formulations that could have better potency or tissue penetration, and antibody cocktails or poly-specific antibodies that might be more robust to viral evolution.^28^ The results of this comparison suggest that if new monoclonal antibody therapies can be developed that effectively neutralise current and future SARS-CoV-2 variants then they could continue to benefit patients admitted to hospital. Viral nucleocapsid antigenaemia is a promising biomarker to guide monoclonal antibody treatment that could aid the development of future monoclonal antibody therapies, but it requires further validation.

Most patients in the RECOVERY sotrovimab comparison were recruited in 2022, and, other than lineage-defining omicron mutations, we identified few mutations conferring sotrovimab resistance in either baseline or follow-up samples. Because of concerns about possible reduced sotrovimab activity against BA.1, a 1 g dose was used in RECOVERY rather than the 500 mg dose tested previously, and this was well tolerated with no new safety concerns. The absence of any measurable effect of sotrovimab on nasal SARS-CoV-2 carriage by day 5 could be related to the early sampling timepoints used, because, even in patients who are seronegative and treated with a well matched monoclonal antibody, a reduction in carriage of viral RNA is mainly apparent from day 7 onwards.^5^ Unlike viral RNA carriage, a large reduction in culturable SARS-CoV-2 can be seen as early as 24 h after monoclonal antibody therapy, but virological testing in RECOVERY did not extend to culture.^29^ The emergence of new viral resistance mutations during sotrovimab treatment is well described, especially in patients who are immunocompromised, but there was little evidence of this in RECOVERY.^30,31^ Only two patients treated with sotrovimab had resistance mutations identified by day 5, although emergent resistance is often only identified at later timepoints, and the detection of resistance was not a principal aim of the trial.

Strengths of this trial include that it was randomised, had broad eligibility criteria, and a large sample size, being the second largest trial of neutralising monoclonal antibody therapy performed in patients admitted to hospital with COVID-19. This trial included baseline characterisation of markers of SARS-CoV-2 immune status and infection, and 1710 (99%) of 1723 patients were followed up for the primary and secondary outcomes. The study has some limitations: the use of serum antigen to define the primary analysis population was prespecified, but this is a novel therapeutic biomarker and there is little existing evidence to support the threshold used to classify patients. The distribution of serum antigen in our population was unimodal with no natural cut-point, so other thresholds could have been selected, and further validation of this threshold would be needed for clinical use. In a larger trial it might have been possible to retrospectively identify an optimal antigen threshold, but this kind of sensitivity analysis would not be robust in our study, as this would require more outcome events. This trial was also not large enough to reliably exclude benefit among patients with a low antigen level, or to exclude differences in treatment effect among specific subgroups of patients based on characteristics such as time since symptom onset, immunodeficiency, or concomitant use of remdesivir. Remdesivir was received by 628 (36%) patients, and it is possible that sotrovimab would have had a greater effect in the absence of concomitant antiviral treatment. The RECOVERY trial was open label, which meant participants and local hospital staff were aware of the assigned treatment. The open-label design could potentially have affected clinical management or the recording of some trial outcomes, although we found no evidence that management differed by treatment allocation (appendix p 61), and the primary and secondary outcomes are unambiguous and were ascertained without bias through linkage to routine health records. Although virological outcomes were included, outcomes did not include viral culture or virological endpoints beyond day 5, and no information on radiological or physiological outcomes was collected. The RECOVERY trial only studied a cohort of patients admitted to hospital who were at high risk of death, therefore, the results might not be directly applicable to the safety and efficacy of treatment in other patient groups, such as patients admitted to hospital who are at lower risk, or those with early infection.

In summary, the results of this randomised trial indicate that many patients who are admitted to hospital with COVID-19 at high risk of death could continue to benefit from monoclonal antibody therapy, and that antigen testing could help to identify these patients. Although no currently available monoclonal antibodies have satisfactory activity against current SARS-CoV-2 variants, these results should inform future monoclonal antibody evaluation and treatment strategies.

## Supplementary Material

Supplementary appendix

## Figures and Tables

**Table 1:** Baseline characteristics

	Patients with a high antigen level		All patients who were randomly assigned	
	Sotrovimab (n=355)	Usual care (n=365)	Sotrovimab (n=828)	Usual care (n=895)
Age, years	72·5 (13·3)	72.1 (13·7)	70·9 (14·2)	70·4 (15·4)
Age group, years				
<70	123 (35%)	141 (39%)	342 (41%)	369 (41%)
≥70 to <80	123 (35%)	121 (33%)	251 (30%)	272 (30%)
≥80	109 (31%)	103 (28%)	235 (28%)	254 (28%)
Sex				
Male	218 (61%)	226 (62%)	490 (59%)	543(61%)
Female	137 (39%)	139 (38%)	338 (41%)	352 (39%)
Race or ethnicity				
White	301 (85%)	333(91%)	706 (85%)	779 (87%)
Black, Asian, and minority ethnic	32 (9%)	16 (4%)	64 (8%)	65 (7%)
Unknown	22 (6%)	16 (4%)	58 (7%)	51 (6%)
Time since symptom onset, days	6 (3–11)	6(3–12)	6 (3–11)	6 (3–11)
Time since admission to hospital, days	2(1–5)	2(1–5)	2(1–5)	2(1–5)
Respiratory support received				
None	43 (12%)	54 (15%)	119 (14%)	137 (15%)
Simple oxygen	226 (64%)	213 (58%)	512 (62%)	557 (62%)
Non-invasive ventilation	71 (20%)	87 (24%)	168 (20%)	169 (19%)
Invasive mechanical ventilation	15 (4%)	11 (3%)	29 (4%)	32 (4%)
Previous diseases				
Diabetes	107 (30%)	84 (23%)	249 (30%)	219 (24%)
Heart disease	119 (34%)	113 (31%)	259 (31%)	272 (30%)
Chronic lung disease	123 (35%)	128 (35%)	327 (39%)	325 (36%)
Tuberculosis	0	1 (<1%)	2 (<1%)	4 (<1%)
HIV	3 (1%)	1 (<1%)	6 (1%)	5 (1%)
Severe liver disease*	6 (2%)	3 (1%)	19 (2%)	16 (2%)
Severe kidney impairment^†^	45 (13%)	41 (11%)	84 (10%)	74 (8%)
Any of the above	242 (68%)	237 (65%)	578 (70%)	602 (67%)
Severely immunocompromised^‡^	112 (32%)	112 (31%)	206 (25%)	208 (23%)
Received a COVID-19 vaccine	296 (83%)	292 (80%)	675 (82%)	714 (80%)
Use of other treatments				
Corticosteroids§	329 (93%)	334 (92%)	755 (91%)	801 (89%)
Remdesivir	144 (41%)	128 (35%)	315 (38%)	313 (35%)
Tocilizumab	66 (19%)	60 (16%)	144 (17%)	137 (15%)
Plan to use tocilizumab within the next 24 h	28 (8%)	33 (9%)	48 (6%)	67 (7%)
Viral load in baseline nose swab, log viral copies per mL	6·1 (4·6–7·0)	6·1 (5·0–7·2)	5·6 (3–7–6·7)	5.6 (3·9–6.8)
Antigen status				
High	355 (100%)	365 (100%)	355 (43%)	365 (41%)
Low	0	0	339 (41%)	378 (42%)
Unknown	0	0	134 (16%)	152 (17%)
Serostatus (anti-nucleocapsid antibodies)				
Positive	62 (17%)	76 (21%)	214 (26%)	240 (27%)
Negative	293 (83%)	289 (79%)	481 (58%)	504 (56%)
Unknown	0	0	133 (16%)	151 (17%)
Serostatus (anti-spike antibodies)				
Positive	252 (71%)	262 (72%)	569 (69%)	610 (68%)
Negative	103 (29%)	103 (28%)	126 (15%)	133 (15%)
Unknown	0	0	133 (16%)	152 (17%)

Data are mean (SD), n (%), or median (IQR). Four female participants who were pregnant were randomly assigned. Race and ethnicity data were collected via linkage to UK NHS records. NHS=National Health Service.

* Defined as requiring ongoing specialist care.

† Defined as estimated glomerular filtration rate <30 mL/min per 1·73 m^2^.

‡ In the opinion of the managing clinician.

§ Including all those who were randomly assigned in the comparison of high-dose versus low-dose steroids.

**Table 2:** Effect of allocation to sotrovimab on key study outcomes in patients with a high antigen level

	Sotrovimab (n=355)	Usual care (n=365)	Rate ratio, risk ratio, or mean difference (95% CI)	p value
**Primary outcome**				
28-day mortality	82 (23%)	106 (29%)	0·75 (0·56 to 0·99)	0·046
**Secondary outcomes**				
Median (IQR) time to being discharged alive, days	13 (7 to >28)	16 (7 to >28)	··	··
Discharged from hospital within 28 days	236 (66%)	226 (62%)	1·12 (0·93 to 1·34)	··
Receipt of invasive mechanical ventilation or death*	82/340 (24%)	102/354 (29%)	0·82 (0·64 to 1·03)	··
Invasive mechanical ventilation	14/340 (4%)	11/354 (3%)	1·71 (0·81 to 3·61)	··
Death	74/340 (22%)	100/354 (28%)	0·74 (0·58 to 0·95)	··
**Subsidiary clinical outcomes**				
Use of ventilation^†^	41/269 (15%)	41/267 (15%)	0·97 (0·66 to 1·44)	··
Non-invasive ventilation	40/269 (15%)	41/267 (15%)	0·95 (0·64 to 1·41)	··
Invasive mechanical ventilation	6/269 (2%)	3/267 (1%)	1.82 (0·47 to 7·11)	··
Successful cessation of invasive mechanical ventilation^‡^	5/15 (33%)	3/11 (27%)	1·07 (0·25 to 4·65)	··
Use of haemodialysis or haemofiltration^§^	12/347 (3%)	6/356 (2%)	1·97 (0·77 to 5·06)	··
**Virological outcomes**				
Baseline-adjusted viral load (log copies per mL) on day 3	4·89 (0·10)	4·94 (0·10)	–0·05 (–0·32 to 0·23)	··
Baseline-adjusted viral load (log copies per mL) on day 5	4·26 (0·11)	4·35 (0·10)	–0.09 (–0·38 to 0·20)	··

Data are n (%) or n/N (%), unless otherwise indicated. Rate ratio for the outcomes of 28-day mortality and hospital discharge, risk ratio for other clinical outcomes, and mean difference for virological outcomes. Estimates of the rate ratio, risk ratio, or mean difference and their 95% CIs are adjusted for age in three categories (<70 years, 70–79 years, and 80 years or older) and ventilation status at randomisation in four categories (none, simple oxygen, non-invasive ventilation, and invasive mechanical ventilation). p values are not shown for the secondary, subsidiary or virological outcomes because the hierarchical testing strategy prespecified in the statistical analysis plan stated that such tests would only be performed if the null hypothesis for the primary outcome of 28-day mortality was rejected in both the antigen positive subgroup and in the whole population.

* Excluding patients receiving invasive mechanical ventilation at randomisation.

† Excluding patients receiving invasive or non-invasive ventilation at randomisation.

‡ Excluding patients not receiving invasive mechanical ventilation at randomisation.

§ Excluding patients receiving renal replacement therapy at randomisation.

## Data Availability

The protocol, consent form, statistical analysis plan, definition and derivation of clinical characteristics and outcomes, training materials, regulatory documents, and other relevant study materials are available online. As described in the protocol, the trial steering committee will facilitate the use of the study data and approval will not be unreasonably withheld. De-identified participant data and a data dictionary will be made available to bona fide researchers registered with an appropriate institution within 3 months of publication. However, the steering committee will need to be satisfied that any proposed publication is of high quality, honours the commitments made to the study participants in the consent documentation and ethical approvals, and is compliant with relevant legal and regulatory requirements (eg, relating to data protection and privacy). The steering committee will have the right to review and comment on any draft manuscripts before publication. Data will be made available in line with the policy and procedures described at: https://www.ndph.ox.ac.uk/data-access. Those wishing to request access should complete the form available at this site.

## Research in context

### Evidence before this study

We searched MEDLINE, Embase, and medRxiv from Sept 1, 2019, up to July 9, 2024, for randomised controlled trials comparing the effects of neutralising monoclonal antibodies versus usual care or placebo in patients admitted to hospital with COVID-19. We used the search terms (“coronavirus infection” OR “COVID” OR “COVID-19” OR “COVID19” OR “2019n-CoV” OR “SARS-COV-2” OR “SARSCoV2” OR “SARS-Cov2”) AND (“sotrovimab”, “S309”, “VIR-7831”, “Xevudy”, “adintrevimab”, “amubarvimab”, “bamlanivimab”, “bebtelovimab”, “casirivimab”, “cilgavimab”, “etesevimab”, “imdevimab”, “regdanvimab”, “romlusevimab”, “tixagevimab”) and validated filters to select for randomised controlled trials. No language restrictions were applied. We identified six trials that tested neutralising monoclonal antibodies in patients admitted to hospital with COVID-19, which recruited patients between May, 2020, and July, 2022 (appendix p 35). In all six trials, most patients were infected with pre-omicron SARS-CoV-2 variants. The RECOVERY casirivimab–imdevimab comparison was the largest trial identified and included six times as many patients who died than the other trials combined. No definite effect of neutralising monoclonal antibodies was seen on 28-day all-cause mortality in analyses of all trial participants (in RECOVERY 943 [19%] of 4839 patients assigned to casirivimab–imdevimab died *vs* 1029 [21%] of 4946 patients assigned to usual care; rate ratio [RR] 0·94, 95% CI 0·86–1·02; p=0·14). In the RECOVERY casirivimab–imdevimab comparison, monoclonal antibody treatment was associated with a large and definite reduction in mortality in patients who had not yet developed a SARS-CoV-2 antibody response at baseline (ie, were seronegative); 396 (24%) of 1633 patients assigned to monoclonal antibody treatment died versus 452 (30%) of 1520 patients assigned to usual care (RR 0·79, 95% CI 0·69–0·91; p=0·0009). Two other trials reported mortality by antibody status at baseline (ACTIV-3/TICO and COV-2066), and a similar benefit of neutralising monoclonal antibody therapy was also observed in over 1000 patients who were seronegative in these trials. Since these three trials were reported, increasing rates of vaccination and previous infection have meant that few patients admitted to hospital are now antibody negative, and from 2022, most neutralising monoclonal antibodies in clinical use have shown substantial losses of in-vitro potency against prevalent omicron variants. One trial, ACTIV-3/TICO, reported outcomes among patients with high (higher than median) blood SARS-CoV-2 nucleocapsid antigen concentration at baseline, although this trial was not powered to detect or rule out a clinically meaningful benefit of treatment in this group. 43 (13%) of 340 patients with high blood antigen levels assigned to tixagevimab–cilgavimab died within 90 days, compared with 51 (15%) of 342 patients assigned to placebo (hazard ratio 0·84; 95% CI 0·56–1·26).

### Added value of this study

To our knowledge, this is the second-largest trial of neutralising monoclonal antibody therapy for patients admitted to hospital with COVID-19. Moreover, this is the first trial directed at patients with omicron infection, and the first performed in a population who were predominantly vaccinated. This RECOVERY comparison evaluated sotrovimab, a monoclonal antibody that retained some in vitro neutralisation activity against omicron variants dominant in 2022–23, but which has minimal in vitro neutralisation activity against several variants that were dominant from 2024. Because most patients admitted to hospital had antibodies from previous vaccination or infection, serum antigen concentrations were used to identify patients who were at high risk of death and might benefit from treatment. In the prespecified analysis population of patients with high serum antigen concentrations at baseline, sotrovimab was associated with a reduction in the primary outcome of 28-day mortality (82 [23%] of 355 patients assigned to sotrovimab died *vs* 106 [29%] of 365 patients assigned to usual care; RR 0·75, 95% CI 0·56–0·99; p=0·046).

### Implications of all the available evidence

Monoclonal antibody therapy could potentially benefit current patients admitted to hospital with COVID-19, including those with previous vaccination and infection with omicron variants. Efficacy appears to be restricted to patients who have not yet mounted an effective immune response to their infection, in whom mortality remains high despite current treatment. However, the emergence of variants resistant to neutralisation by sotrovimab highlights the need for newer monoclonal antibody therapies with reliable and durable neutralising activity against current and future SARS-CoV-2 variants. Measurement of serum viral antigen concentrations offers a promising approach to targeting treatment that could facilitate future monoclonal development, but this approach needs further validation.
